# Evaluation of the Sofia S. pneumoniae FIA for Detection of Pneumococcal Antigen in Patients with Bloodstream Infection

**DOI:** 10.1128/JCM.01535-18

**Published:** 2019-07-26

**Authors:** Emma Olofsson, Volkan Özenci, Simon Athlin

**Affiliations:** a Faculty of Medicine and Health, Örebro University, Örebro, Sweden; b Department of Clinical Microbiology F 72, Karolinska University Hospital Huddinge, Stockholm, Sweden; c Division of Clinical Microbiology, Department of Laboratory Medicine, Karolinska Institutet, Stockholm, Sweden; d Department of Infectious Diseases, Faculty of Medicine and Health, Örebro University, Örebro, Sweden; University of Tennessee at Knoxville

**Keywords:** *Streptococcus pneumoniae*, antigen specificity, community-acquired infections, pneumococcus, pneumonia, urinary antigen test

## Abstract

The usefulness of pneumococcal urinary antigen tests (UATs) in severe pneumococcal infection relies heavily on the performance in bacteremic patients. Fluorescence technology and automatic reading of test results may improve UAT performance. We evaluated the automatically read Sofia S. pneumoniae FIA for diagnosing pneumococcal bloodstream infection (BSI) in hospitalized adult patients. First, the Sofia FIA was evaluated on 97 patients with pneumococcal (*n* = 47) and nonpneumococcal (*n* = 50) BSI and compared with results by the visually read BinaxNOW S. pneumoniae immunochromatographic test (ICT) and ImmuView S. pneumoniae and Legionella pneumophila ICT. In four cases (4.1%), the Sofia FIA showed invalid test results, three of which showed invalid results by the ImmuView ICT previously. Based on 93 valid cases, the Sofia FIA showed similar sensitivity (for both comparisons: 68% versus 62%; *P* = 0.45) and specificity (for both comparisons: 91% versus 93%; *P* = 1.00) as the visually read UATs. Second, the Sofia FIA was prospectively evaluated on 82 consecutive nonfrozen urine samples, detecting pneumococcal antigen in 10 of 14 (sensitivity, 71%) pneumococcal BSI patients, similarly to the visually and automatically read BinaxNOW ICT (both 12 of 14; sensitivity, 86%; *P* = 0.50). Of five nonpneumococcal BSI cases, the Sofia FIA showed an invalid test result in one case, but no positive UAT results were obtained. Thus, the sensitivity and specificity of the Sofia FIA were similar to the performance rates of other UATs in patients with BSI, but invalid test results are of concern for the usefulness in pneumococcal BSI.

## Abstract (precis)

The usefulness of pneumococcal urinary antigen tests (UATs) in severe pneumococcal infection relies heavily on the performance in bacteremic patients. Fluorescence technology and automatic reading of test results may improve UAT performance. We evaluated the automatically read Sofia S. pneumoniae FIA for diagnosing pneumococcal bloodstream infection (BSI) in hospitalized adult patients.

## INTRODUCTION

International guidelines recommend the use of urinary antigen tests (UATs) for rapid identification of pneumococcal etiology in adult pneumonia (1, 2). A positive UAT may enhance the diagnostic yield and supports narrow-spectrum antibiotic therapy, but the clinical usefulness of UATs has been questioned (3–6). Because targeted treatment in severe pneumococcal infection relies heavily on diagnostic methods with high performance, the development of technologies with improved sensitivity is desirable. We previously reported that novel UATs have increased positivity rates in patients with bloodstream infections (BSI). However, the assays suffered from impaired specificity and validity in BSI (7, 8).

The Sofia Streptococcus pneumoniae fluorescence immunoassay (FIA; Quidel, USA) detects pneumococcal cell wall polysaccharides (CWPS) in urine by using immunofluorescence-based lateral-flow technology and received the CE mark in 2016. Test results are interpreted automatically by the Sofia Analyzer after 10 min and may be positive, negative, or invalid. For invalid test results, a new test is recommended with a new patient sample and a new test cassette. When the test was compared with the BinaxNOW S. pneumoniae immunochromatographic test (BinaxNOW ICT; Abbott, USA) for diagnosing pneumococcal pneumonia, the automatically read Sofia FIA detected 33% more cases than the visually read BinaxNOW ICT (9). It was suggested that the improved sensitivity yield derived from the automatically interpretation of the test, which avoids false-negative results in cases of weak signals.

In this study, we evaluated the automatically read Sofia FIA on urine samples from patients with BSI and compared the results with those previously obtained by the visually read BinaxNOW ICT and ImmuView S. pneumoniae and L. pneumophila ICT (ImmuView ICT; SSI Diagnostica A/S, Denmark). In addition, we evaluated the Sofia FIA for diagnosing pneumococcal BSI in consecutive nonfrozen samples compared to the visually and automatically read BinaxNOW ICT.

(Preliminary data were presented at the 11th International Symposium on Pneumococci and Pneumococcal Diseases [ISPPD-11], Melbourne, Australia, 15 to 19 April 2018 [10].)

## MATERIALS AND METHODS

First, blood and urine samples were collected simultaneously from adult patients (≥18 years) with suspected BSI between 2013 and 2016 at Örebro University Hospital and Karolinska University Hospital, Huddinge. Blood cultures (BCs) were processed according to standard methods (11). Cases with coagulase-negative staphylococci in only one BC bottle were excluded. All urine samples were frozen at −20°C until they were thawed in 2016 for a performance study of the ImmuView ICT in comparison with the BinaxNOW ICT and the BinaxNOW L. pneumophila ICT (Alere) for detection of pneumococcal and *Legionella* antigen in urine (8). After visual reading of test results, the samples were refrozen at −20°C.

In April 2017, the samples were thawed at room temperature or in a water bath and vortexed for 5 s, but not concentrated or boiled prior to testing, and then tested under blinded conditions with the Sofia FIA in a randomized order and according to the manufacturer’s guidelines. The test results were automatically interpreted by the Sofia Analyzer and compared to the visually read results for pneumococcal antigen detection by the BinaxNOW and ImmuView ICTs previously obtained (8). For invalid test results, the same urine sample was retested with another test cassette, and the sample was analyzed for increased levels of glucose, erythrocytes, and leucocytes by Multistix 7 (Siemens Healthcare Diagnostics, USA).

Second, the Sofia FIA was prospectively evaluated on consecutive nonfrozen urine samples in comparison with the visually and automatically read BinaxNOW ICT. Consecutive samples from adult patients (≥18 years) were tested between April and June 2017 at the Department of Laboratory Medicine, Örebro University Hospital, Sweden. Inclusion criteria were urine samples collected in the clinical routine work for (i) pneumococcal antigen detection or (ii) cultivation of urinary tract pathogens if the patient was positive for S. pneumoniae in BC simultaneously. One sample per patient was analyzed. BCs were processed according to standard methods (11). Cases with coagulase-negative staphylococci in only one BC bottle were excluded. After recording the patient’s age and sex, all samples were randomized and tested under blinded conditions according to the manufacturers’ instructions. The Sofia FIA was automatically read by the Sofia Analyzer, while the BinaxNOW ICT was read visually as well as automatically by the Alere Reader. The samples were not frozen or concentrated before testing. BC was used as reference standard for evaluation of the UAT performance. Samples yielding invalid or discordant UAT results were retested. Positive cultures for S. pneumoniae in nasopharynx (NP) were recorded in cases with invalid test results and positive UAT results in BC-negative cases.

### Statistics.

The interassay agreement between test results was calculated using the first test result or, if an invalid test, any valid result after retesting and was estimated by calculating Cohen’s unweighted kappa coefficient (*κ*) (12). For comparison of sensitivity and specificity rates, the McNemar’s test was used with a two-tailed *P* value of <0.05 considered statistically significant. A confidence interval (CI) of 95% was used for statistical precision. The statistical analyses were performed with a statistical software package (SPSS for windows, version 22.0).

## RESULTS

The Sofia FIA was performed on urine samples from 97 patients (median age, 74 years [range, 18 to 96 years]; female, 43%) with pneumococcal (*n* = 47) or nonpneumococcal (*n* = 50) BSI and compared to previously obtained results by the visually read BinaxNOW and ImmuView ICTs (8). In four (4.1%) cases, the Sofia FIA showed invalid test results, three (Escherichia coli, *n* = 2; Proteus mirabilis, *n* = 1) of which were invalid by the ImmuView ICT previously and contained high levels of erythrocytes (≥80 erythrocytes/μl), leucocytes (≥70 leucocytes/μl), and/or glucose (28 mmol/liter). In one case (Enterococcus faecalis) containing high levels of erythrocytes (80/μl), the Sofia FIA was invalid alone. In one of the invalid cases (P. mirabilis), the Sofia FIA was positive by at retesting.

Based on 93 valid test results by all UATs, the agreement was good between the Sofia FIA and the BinaxNOW and ImmuView ICTs (both *κ* = 0.77; CI, 0.63 to 0.90). The UAT results are presented in Table S1 in the supplemental material. In 13 cases (pneumococcal, *n* = 10; nonpneumococcal, *n* = 3), discordant test results were obtained (Table 1). The sensitivity of the Sofia FIA was 68% (32/47; CI, 54% to 80%) compared to 62% (both 29/47; CI, 47% to 74%; *P* = 0.45) for the BinaxNOW and ImmuView ICTs, and the specificity was 91% (42/46; CI, 80% to 97%) compared to 93% (both 43/46; CI, 83% to 98%; *P* = 1.00) for the BinaxNOW and ImmuView ICTs.

**TABLE 1:** Thirteen discordant test results by the Sofia S. pneumoniae FIA, the BinaxNOW S. pneumoniae ICT, and the ImmuView S. pneumoniae and L. pneumophila ICT in 93 patients with bloodstream infection^*a*^

BC^*b*^ result	*n*	Test result		
		Sofia FIA (automatic)	BinaxNOW ICT (visual)	ImmuView ICT (visual)
Streptococcus pneumoniae	3	+	−	−
Streptococcus pneumoniae	2	+	+	−
Streptococcus pneumoniae	2	+	−	+
Streptococcus pneumoniae	1	−	+	+
Streptococcus pneumoniae	1	−	+	−
Streptococcus pneumoniae	1	−	−	+
Bacillus cereus	1	+	−	−
Streptococcus mitis	1	+	−	−
Enterococcus faecalis	1	−	+	+

a FIA, fluorescence immunoassay; ICT, immunochromatographic test.

b BC, blood culture.

Second, the Sofia FIA was performed on 82 consecutive nonfrozen urine samples (median age, 71 years [range, 18 to 98 years]; female, 51%) and compared to the visually and automatically read BinaxNOW ICT. BC was performed in 79 (96%) cases, 19 (24%) of which had pneumococcal (*n* = 14) or nonpneumococcal (*n* = 5) BSI. In one nonpneumococcal BSI case, the Sofia FIA was invalid, also at retesting, but the BinaxNOW ICT was negative visually and automatically. The patient had E. coli in BC but negative NP culture and had no interfering substances in urine.

Based on 78 cases with BC performed and valid test results, the Sofia FIA demonstrated a very good overall agreement with the visually (*κ* = 0.85; CI, 0.71 to 0.99) and automatically (*κ* = 0.82; CI, 0.67 to 0.97) interpreted BinaxNOW ICT. The UAT results are presented in Table S2. Nine cases with positive UAT results were negative in BC, four of which were positive for S. pneumoniae in NP culture. Three of the culture positive cases were positive by the Sofia FIA as well as the BinaxNOW ICT visually and automatically, and one was positive by the BinaxNOW ICT visually alone. Six cases yielded discordant UAT results, one of which was positive for S. pneumoniae in NP culture. By retesting urine samples with discordant results, the initial results were repeated in three cases, and altered results were obtained in three cases (Table 2). Calculated on pneumococcal BSI cases (*n* = 14), the sensitivity was 71% (10/14; CI, 45% to 88%) for the Sofia FIA and 86% (both 12/14; CI, 60% to 96%; *P* = 0.50) for the BinaxNOW ICT visually and automatically. All nonpneumococcal BSI cases yielded negative UAT results; however, the number of valid cases (*n* = 4) was too small for specificity calculation.

**TABLE 2:** Six discordant test results by the Sofia S. pneumoniae FIA and the BinaxNOW S. pneumoniae ICT in 78 patients consecutively tested for pneumococcal antigen in clinical routine^*a*^

BC^*b*^ result	*n*	Sofia FIA result (automatic)		BinaxNOW ICT result			
				Visual		Automatic	
		Test	Retest	Test	Retest	Test	Retest
Streptococcus pneumoniae	2	−	−	+	+	+	+
Negative	1	−	−	+	+	+	+
Negative^*c*^	1	−	−	−	−	+	+
Negative	1	+	+	−	−	+	−
Negative	1	−	+	−	+	+	+

a FIA, fluorescence immunoassay; ICT, immunochromatographic test.

b BC, blood culture.

c The patient was positive for S. pneumoniae in nasopharyngeal culture.

## DISCUSSION

Fluorescence detection and automatic reading of UAT results may enhance test sensitivity and remove user dependent variation, but validity and specificity of test results is of concern in severe pneumococcal infection. In this study, we compared the automatically interpreted Sofia FIA with the visually read BinaxNOW and ImmuView ICTs for detection of pneumococcal antigen in patients with BSI but observed no enhanced sensitivity by automatic reading. In addition, we observed similar sensitivities of the Sofia FIA and the visually and automatically read BinaxNOW ICT for diagnosing pneumococcal BSI in consecutively tested urine samples. However, we recorded invalid test results on urine samples containing interfering substances, similarly to the ImmuView ICT previously described (8).

We observed good agreement between the Sofia FIA and the BinaxNOW ICT (κ = 0.77) and the ImmuView ICT (κ = 0.77), yielding similar sensitivities in patients with BSI (for both comparisons, 68% versus 62%, *P* = 0.45). Also, the agreement was very good between the Sofia FIA and the visually (*κ* = 0.85) and automatically (*κ* = 0.82) read BinaxNOW ICT in consecutive nonfrozen samples, yielding similar sensitivities in pneumococcal BSI cases (71% versus 86%; *P = *0.50). Burgos et al. recently observed good agreement (*κ* = 0.81) between the Sofia FIA and the visually read BinaxNOW ICT in 219 patients with community-acquired pneumonia (CAP) and slightly higher sensitivity of the Sofia FIA in 14 cases with proven pneumococcal etiology (79% versus 50%; *P = *0.12) (9). Unfortunately, the positivity rate in pneumococcal BSI cases was not defined. In a study by Vicente et al., the Sofia FIA was positive in four of five (75%) bacteremic patients, with an overall sensitivity of 77% in pneumococcal CAP, but the definition included cases based on positive BinaxNOW ICT results alone, which may overestimate the performance (13).

As in previous studies, the UATs failed to detect pneumococcal antigen in approximately one-third of pneumococcal BSI cases (14). The test sensitivity in BSI is of concern, since a positive UAT result is associated with poor clinical outcome (15). Low CWPS load in bloodstream and impaired renal function are probable explanations to negative results (15, 16). By concentrating the urine, the sensitivity of pneumococcal antigen detection may be improved (17, 18) but is not recommended by the UAT manufacturers and was not performed in this study. In addition, CWPS may bind to antibodies to form immune complexes in blood and urine (19) and therefore will not be detected in low concentrations, but false-negative results may depend on clinical factors as well (20).

Fluorescence technology appears to improve the sensitivity of lateral-flow tests for the detection of *Legionella* in urine compared to colorimetric detection but may increase the risk of false-positive test results (21). Likewise, pneumococcal UATs have shown false positivity in BSI patients previously, mainly caused by cross-reactivity to alpha-hemolytic streptococci but also to Gram-negative bacteria (7, 22). In this study, the specificity of the Sofia FIA (91%) was similar to that of the BinaxNOW and ImmuView ICTs (both, 93%; *P* = 1.00), indicating that fluorescence technology is not inferior to colorimetric detection regarding specificity. When evaluated on nonfrozen samples, both the Sofia FIA and the BinaxNOW ICT were negative in nonpneumococcal BSI cases. Burgos et al. observed equal specificities of 79% of the Sofia FIA and the BinaxNOW ICT, as both methods showed false-positive results in 3 of 14 nonpneumococcal CAP cases (9). However, the three cases were BC positive for different bacteria than the false-positive BSI cases in our study, suggesting that false positivity may be due to other factors than cross-reactivity between bacterial species alone. Furthermore, Vicente et al. observed a specificity of 87% for the Sofia FIA in nonpneumococcal CAP patients, one of which was BC positive for Streptococcus agalactiae, but the specificity increased to 92% by heating the samples (13). Preheating of urine is not recommended by the UAT manufacturers and was not performed in our study but may be of additional value to reduce false positivity.

The Sofia FIA yielded invalid test results in four cases (4.1%) when evaluated on frozen samples, three of which had shown invalid results by the ImmuView ICT but not the BinaxNOW ICT previously (8). In addition, the Sofia FIA was invalid in a patient with E. coli bacteremia when evaluated on nonfrozen samples. High viscosity and/or interfering components, such as high levels of erythrocytes, leucocytes, and glucose, seem to have a negative impact on test performance. The impact of heating, filtration, or centrifugation on the validity of the Sofia FIA was not evaluated in this study, since this is not recommended by the manufacturer, but should be considered in order to remove interfering substances (23). However, heating the samples did not improve validity of the ImmuView in our previous study, indicating that additional sample preparation is needed in patients with cloudy urine (8).

The major limitation of the study was that we used urine samples which had been repeatedly frozen and thawed prior to performing the Sofia FIA. Freezing of urine samples before testing is an accepted sample preparation process in evaluation studies of UATs, since pneumococcal polysaccharides are considered temperature stable (17, 24, 25). In a study by Saukkoriipi et al., the performance of BinaxNOW ICT was similar when using frozen unconcentrated urine samples after thawing and when using fresh urine (26). In this study, the freezing procedure had probably no impact on the performance of the Sofia FIA, since there was a high proportion of agreement between test results of the UATs.

In conclusion, the automatically read Sofia FIA showed similar sensitivity and specificity as other UATs for the detection of pneumococcal antigen in patients with BSI. The test showed invalid results when applied on urine samples containing interfering substances, similarly as described previously for the ImmuView ICT. Further studies analyzing the underlying reasons for false-positive and invalid test results are needed.

## Supplementary Material

Supplemental file 1
