# Supplementary material for: Evaluation of the Sofia S. pneumoniae FIA for Detection of Pneumococcal Antigen in Patients with Bloodstream Infection
Source: J Clin Microbiol. 2019 Jul 26;57(8):e01535-18. doi: 10.1128/JCM.01535-18 (PMC6663912; doi:10.1128/JCM.01535-18)
Supplement: Supplemental file 1 [file JCM.01535-18-s0001.pdf]

Supplementary Table 1S Results of the Sofia *S. pneumoniae* FIA, the BinaxNOW *S. pneumoniae* ICT and the ImmuView *S. pneumoniae* and *L. pneumophila* ICT in 93 patients with bloodstream infection

BC result	*n*	Sofia FIA	BinaxNOW ICT	ImmuView ICT
		Automatic	Visual	Visual
Streptococcus pneumoniae	47	32	29	29
Escherichia coli	4	1	1	1
Klebsiella pneumoniae	3	0	0	0
Pseudomonas aeruginosa	3	0	0	0
Staphylococcus aureus	3	0	0	0
Streptococcus mitis	3	1	0	0
Enterococcus faecalis	2	0	1	1
Proteus mirabilis	2	0	0	0
Staphylococcus epidermidis	2	1	1	1
Other[a]	23	1[b]	0	0

BC, blood culture; FIA, fluorescence immunoassay; ICT, immunochromatographic test.

[a] One of each: *Bacillus cereus, Bacteroides fragilis, Clostridium paraputrificum, Enterococcus cloacae, Granulicatella adiacens,* Group A streptococcus, Group B streptococcus, Group G streptococcus, *K. oxytoca , Listeria monocytogenes, Morganella morganii, Salmonella typhimur, Streptococcus anginosus, Streptococcus bovis, Streptococcus salivarius, Clostridium perfringens* + *E. coli, E. faecalis* + α-hemolytic streptococcus, *E. faecalis* + *Streptococcus agalactiae, E. faecalis* + *P. mirabilis, E. faecium* + *P. aeruginosa, C. perfringens* + *E. faecium* + *S. epidermidis, C. perfringens* + *K. pneumoniae* + *S. anginosus* and *S. epidermidis* + *S. salivarius* + *S. mitis.*

[b] Positive for *B. cereus* in BC.

Supplementary Table 2S Results of the Sofia *S. pneumoniae* FIA in comparison with the BinaxNOW *S. pneumoniae* ICT in 78 patients consecutively tested for pneumococcal antigen in clinical routine

BC result	*n*	Sofia FIA	BinaxNOW ICT	BinaxNOW ICT
		Automatic	Visual	Automatic
Streptococcus pneumoniae	14	10	12	12
Escherichia coli	1	0	0	0
Group G streptococcus	1	0	0	0
Staphylococcus hominis	1	0	0	0
Staphylococcus aureus + *Streptococcus salivarius*	1	0	0	0
Negative	60[a]	6	6	9

BC, blood culture; FIA, fluorescence immunoassay; ICT, immunochromatographic test.

[a] Missing data for the automatically read BinaxNOW ICT in one case.

The UAT results are presented as recorded before retesting of discordant test results.
